# *Bacillus licheniformis* BlaR1 L3 Loop Is a Zinc Metalloprotease Activated by Self-Proteolysis

**DOI:** 10.1371/journal.pone.0036400

**Published:** 2012-05-18

**Authors:** Stéphanie Berzigotti, Kamal Benlafya, Jérémy Sépulchre, Ana Amoroso, Bernard Joris

**Affiliations:** Centre for Protein Engineering, Department of Life Sciences, University of Liège, Liège, Belgium; Griffith University, Australia

## Abstract

In *Bacillus licheniformis* 749/I, BlaP β-lactamase is induced by the presence of a β-lactam antibiotic outside the cell. The first step in the induction mechanism is the detection of the antibiotic by the membrane-bound penicillin receptor BlaR1 that is composed of two functional domains: a carboxy-terminal domain exposed outside the cell, which acts as a penicillin sensor, and an amino-terminal domain anchored to the cytoplasmic membrane, which works as a transducer-transmitter. The acylation of BlaR1 sensor domain by the antibiotic generates an intramolecular signal that leads to the activation of the L3 cytoplasmic loop of the transmitter by a single-point cleavage. The exact mechanism of L3 activation and the nature of the secondary cytoplasmic signal launched by the activated transmitter remain unknown. However, these two events seem to be linked to the presence of a HEXXH zinc binding motif of neutral zinc metallopeptidases. By different experimental approaches, we demonstrated that the L3 loop binds zinc ion, belongs to Gluzincin metallopeptidase superfamily and is activated by self-proteolysis.

## Introduction

During evolution, the most common resistance mechanism acquired by eubacteria to resist β-lactam antibiotic action is the production of a β-lactamase that degrades these antibiotics by opening their β-lactam ring [1], [2]. The cleaved antibiotic is then unable to acylate its membrane-bound D,D-peptidase targets, which are involved in the peptidoglycan cross-linking or remodeling. These enzymes inhibited by β-lactam antibiotics are named penicillin-binding proteins (PBPs).

In *Bacillus licheniformis* and *Staphylococcus aureus*, the production of an inducible class A β-lactamase, BlaP and BlaZ respectively, is regulated by the BlaI repressor that maintains β-lactamase production at a low level in the absence of a β-lactam antibiotic outside the cell. In presence of a β-lactam antibiotic at sublethal concentration, the BlaI repressor is inactivated by a protein relay including the membrane-bound penicillin-sensory transducer BlaR1 and the BlaR2 protein, yet to be identified [3], [4], [5]. The regulatory genes, *blaI* and *blaR*1 are located downstream the *blaP*/*blaZ* genes and are divergently transcribed as a polycistronic mRNA [6].

In *S. aureus*, another related mechanism that mediates resistance to β-lactam antibiotics has been described. It involves the induction of the low-affinity PBP2a or MecA capable to replace the inhibited constitutive PBPs [7]. The *mecA* expression is under the control of MecI and MecR proteins that are homologous to *S. aureus* and *B. licheniformis* BlaI and BlaR1, and play the same role. Furthermore, MecI and BlaI from *S. aureus* are interchangeable, but BlaR1 and MecR are not [8], [9].

**Figure 1:**
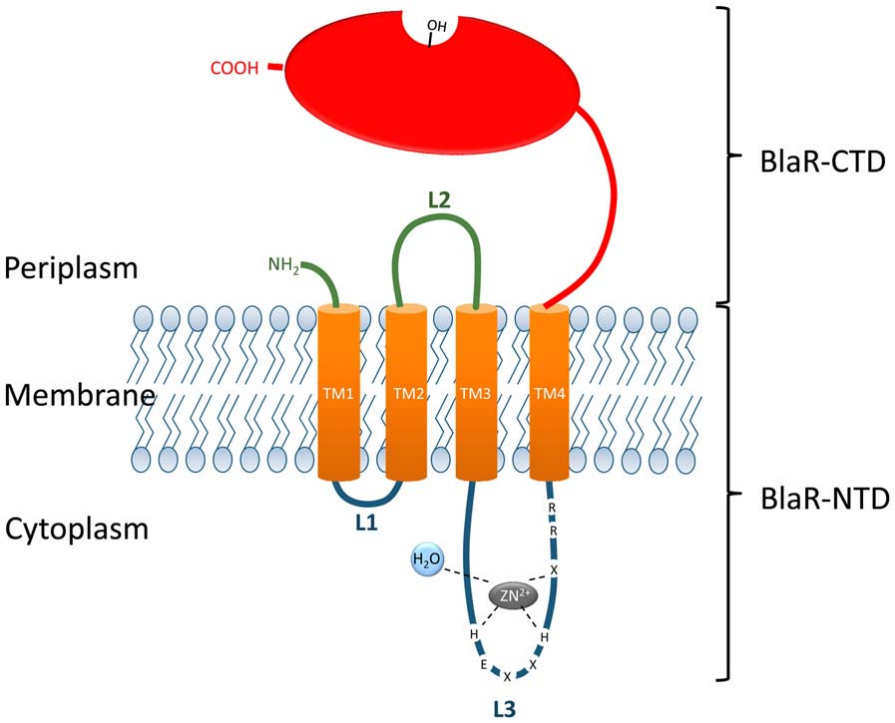
Membrane topology of the penicillin-sensory transducer, BlaR1. This receptor contains two domains, an extracellular domain: BlaR-CTD and a transmembrane domain: BlaR-NTD. BlaR-CTD exhibits the three motifs of the penicillin binding protein family (S*^402^TYK, Y^476^GN, K^539^TG, where S^402^ is the active serine). BlaR-NTD includes four transmembrane segments (TM1, TM2, TM3, TM4) connected by three loops (L1, L2, L3). The cytoplasmic L3 loop contains the H^212^EXXH motif, characteristic to zinc-metalloproteases.

**Figure 2:**
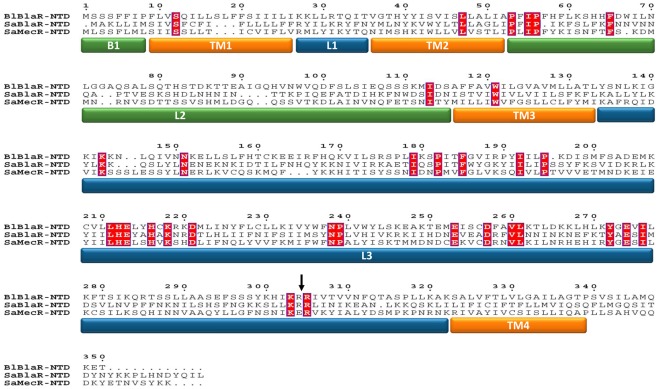
Sequence alignment of the amino-terminal domain of the proteins *B. licheniformis* 749/I BlaR1 (BlBlaR-NTD) and *S. aureus* RN4220 BlaR1 (SaBlaR-NTD) and *S. aureus* MRSA252 MecR (SaMecR-NTD). The conserved residues are highlighted in red and the loop and transmembrane segments are marked: the transmembrane segments (TM1 to 4) in orange; the extracellular loops B1 (M^1^-P^8^) and L2 (P^53^-S^115^) in green and the cytoplasmic loops L1 (K^27^-T^35^) and L3 (Y^134^-K^322^) in blue. The site of cleavage, situated between the R^293^ and R^294^ in *S. aureus* BlaR1, is indicated by an arrow.

The BlaR1 and MecR membrane-bound penicillin-sensory transducers are composed of two domains, an amino-terminal domain (BlaR/MecR-NTD) anchored into the cytoplasmic membrane and a carboxy-terminal domain (BlaR/MecR-CTD) exposed outside the cell. This latter domain contains in its primary structure, the conserved motifs of the serine-penicillin recognizing enzymes (SPRE) and acts as a penicillin sensor [10], [11]. The BlaR-CTD from *B. licheniformis* and from *S. aureus* can be produced as soluble domains and their crystal structures exhibit the conserved SPRE 3D-fold [12], [13], [14]. The membrane topology of *B. licheniformis* BlaR-NTD (Met^1^-Pro^339^) has been experimentally determined: it contains four transmembrane segments (TM1, TM2, TM3, TM4) connected by three loops named L1 (Lys^27^-Thr^35^), L2 (Pro^53^-Ser^115^) and L3 (Tyr^134^-Arg^322^). L1 and L3 are cytoplasmic, whereas L2 is exposed outside the cell (Figure 1) [15]. Although very little is known concerning the mechanism of signal transduction by BlaR1/MecR, the first step is the acylation of BlaR/MecR-CTD by the β-lactam antibiotic outside the cell. The acylation of this domain by the antibiotic causes a modification of the interaction between BlaR-CTD and L2 and gives rise to the activation of the L3 cytoplasmic loop via a conformation change of the four transmembrane segments. The activated L3 would launch a cytoplasmic signal whose target is BlaI/MecI [10], [15], [16], [17], [18]. In this signaling pathway, L2 and the four transmembrane segments would act as a signal transducer and L3 as a cytoplasmic signal amplifier. In *S. aureus*, the BlaR1 L3 activation results in its single-point cleavage between the Arg^293^ and Arg^294^ residues [19]. This cleavage site is conserved in *B. licheniformis* BlaR1 and partially conserved in *S. aureus* MecR (Figure 2). In *S. aureus*, the activated L3 loop generates a cytoplasmic signal that leads to the inactivation of the BlaI repressor by proteolysis whereas in *B. licheniformis* it has been demonstrated that BlaI is not inactivated by proteolysis but by the presence of a coactivator coming from peptidoglycan turnover [20]. Regardless of the route of BlaI inactivation, activated L3 acts as a protease cleaving either BlaI or a pro-coactivator [20], [21], [22]. The hypothesis of L3 protease activity is supported by the presence within the BlaR1/MecR L3 primary structures, of a HEXXH motif characteristic of the zinc-binding signature of the neutral zinc metallopeptidases (M4 superfamily) such as thermolysin (Figure 1) [23]. In *S. aureus* BlaR1, the mutation of the conserved glutamic acid residue to alanine gives rise to a non-inducible β-lactamase phenotype [19].

In this paper, we performed site-directed mutagenesis experiments combined with zinc-blot, Western blot and β-lactamase induction analyses to demonstrate that the cytoplasmic BlaR1 L3 loop belongs to Gluzincin metallopeptidase superfamily and that its cleavage during BlaR1 activation occurs by self-proteolysis.

## Results

### L3 Conserved Residues

A multiple sequence alignment of BlaR1/MecR N-terminal domains of *B. licheniformis* and *S. aureus* (Figure 2) revealed 32 strictly conserved residues in the three aligned sequences corresponding to 9.4% of identity when the length of *B. licheniformis* BlaR-NTD sequence is used as reference ([34/339]×100 = 9.4%). Out of these 32 conserved residues, 24 are located within the L3 loops ([24/187]×100 = 12.8%) highlighting the importance of this conserved loop for intracellular signaling. Conserved residues include the zinc-binding signature HEXXH of neutral zinc metalloproteases. Thermolysin is considered as the canonical enzyme of this protease family, its catalytically essential zinc ion is coordinated by the two histidine residues of the motif (H^142^EXXH, mature thermolysin numbering), the E^166^ and a water molecule. The catalytic mechanism involves a general base mechanism including E^143^ of the H^142^EXXH motif and the protonated H^231^ [24]. Within the strictly conserved residues present in L3 loop consensus, 4 acidic residues (D: 2 and E: 2) could play the role of the third zinc ligand or could be involved in the general base relay. No third conserved histidine residue able to play the catalytic role of H^231^ in thermolysin is present in the L3 loop. The best candidate for this role is a conserved tyrosine residue (Y^272^ in *B. licheniformis* BlaR-NTD).

### Conserved L3 Residues Important for Signal Transduction or Zn Binding

**Figure 3:**
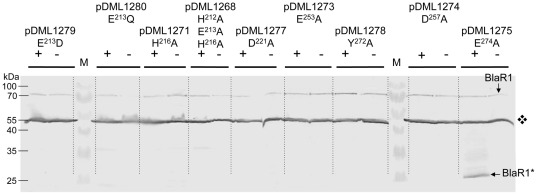
Western blot analysis of membrane-bound BlaR1 produced by *B. subtilis* strains carrying plasmids harboring *blaR*1 mutants. Membrane proteins from induced (+) or uninduced (-) cultures were separatedon on SDS-PAGE. Purified BlaR-CTD antibodies were used for the Western blotting. Pre-stained protein molecular weight markers were used (M). The 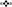 pinpoints an intense and non-specific band (for details see Figure 6). BlaR1 and BlaR1* point out, respectively, the full size and the activated *B. licheniformis* BlaR1 receptor. Except for mutant E^274^A, all other mutants exhibit non-inducible β-lactamase phenotype. The E^274^A mutant has the same profile as the wild-type (to compare see Figure 6). For details see Tables 1 and 2 and Experimental procedures.

To highlight if the conserved E, D, H and Y residues are important for the zinc binding or for the catalytic activity in the activated L3 loop, the mutants listed in Table 1 were constructed. The resulting plasmids (pDML1268-78, Table 2) were introduced into *B. subtilis* 168 and the recombinant resulting strains tested for their ability to induce BlaP β-lactamase in presence of the inducer. In all cases, the mutated receptor expression in non-induced or induced membranes has been detected by Western blotting analysis (Figure 3) showing that the introduced mutations do not lead to the production of an unstable, proteolysis susceptible receptor.

**Table 1:** Induction of BlaPβ-lactamase of the BlaR1 L3 loop mutants.

	Mutation	Plasmids	Induction factor* (3h)
*Wild type*		pDML995	37.8±1.5
*Mutants in H^212^EXXH motif*	H^212^A/E^213^A/H^216^A	pDML1268	1.6±0.1
	E^213^A	pDML1269	1.2±0.1
	H^212^A/H^216^A	pDML1270	1.2±0.1
	H^216^A	pDML1271	1.27±0.01
	E^213^D	pDML1279	1.07±0.02
	E^213^Q	pDML1280	1.6±0.2
*Mutants in putative Zn^++^ ligands*	D^221^A	pDML1277	1.2±0.1
	E^253^A	pDML1273	1.2±0.3
	D^257^A	pDML1274	1.9±0.1
	E^274^A	pDML1275	33.9±0.9
*Mutants in putative general base*	Y^ 272^A	pDML1278	1.1±0.1
			
*Mutants in R^304^R cleavage site*	R^304^A/R^305^A	pDML3045	7.6±1.7

* The induction factor corresponds to the ratio between the β-lactamase quantity/A^600^ for induced culture and the β-lactamase quantity/A^600^ for uninduced culture.

**Table 2:** Bacterial strains and plasmids used in this study.

Strain or plasmid	Relevant characteristic(s)	Source
*Bacillus licheniformis* 749/I	β-lactamase inducible strain, wild-type	
*Bacillus subtilis 168*	Recipient cell for *E. coli*/*Bacillus* shuttle vectors	ATCC 23857
*Escherichia coli* GI724	Host cell for heterologous expression of BlaR1 L3, F-, lamda-, *lac*PL8, *amp*C::Ptrp*cl*	Invitrogen
pUCBM20	Cloning plasmid, Ap^r^	Boehringer
pCR-Script^TM^SK(+)	Cloning vector for PCR fragments, Ap^r^	Stratagene
pLEX	Expression vector from inducible P-lambda promoter, Ap^r^	Novagen
pDML995	A derivative of the *Bacillus*/*E. coli* shuttle vector pMK4 [39] carrying the wild-type *B. licheniformis* 749I *bla*divergon, Ap^r^ in *E.coli* and Cm^r^ in *Bacillus*	A. Brans (unpublished)
pDML1251	pUCBM20 derivative carrying the *Sst*I/*Eco*RI*blaR*1 fragment	This study
pDML1255	pDML1251 derivative carrying BlaR1H^212^A/E^213^A/H^216^A mutation	This study
pDML1256	pDML1251 derivative carrying BlaR1 E^213^A mutation	This study
pDML1257	pDML1251 derivative carrying BlaR1 H^212^A/H^216^A mutation	This study
pDML1258	pDML1251 derivative carrying BlaR1 H^216^A mutation	This study
pDML1260	pDML1251 derivative carrying BlaR1 E^253^A mutation	This study
pDML1261	pDML1251 derivative carrying BlaR1 D^257^A mutation	This study
pDML1262	pDML1251 derivative carrying BlaR1 E^274^A mutation	This study
pDML1264	pDML1251 derivative carrying BlaR1 D^221^A mutation	This study
pDML1265	pDML1251 derivative carrying BlaR1 Y^272^A mutation	This study
pDML1266	pDML1251 derivative carrying BlaR1 E^213^D mutation	This study
pDML1267	pDML1251 derivative carrying BlaR1 E^213^Q mutation	This study
pDML1268	pDML995 derivative carrying BlaR1 H^212^A/E^213^A/H^216^A mutation	This study
pDML1269	pDML995 derivative carrying BlaR1 E^213^A mutation	This study
pDML1270	pDML995 derivative carrying BlaR1 H^212^A/H^216^A mutation	This study
pDML1271	pDML995 derivative carrying BlaR1 H^216^A mutation	This study
pDML1273	pDML995 derivative carrying BlaR1 E^253^A mutation	This study
pDML1274	pDML995 derivative carrying BlaR1 D^257^Amutation	This study
pDML1275	pDML995 derivative carrying BlaR1 E^274^A mutation	This study
pDML1277	pDML995 derivative carrying BlaR1 D^221^A mutation	This study
pDML1278	pDML995 derivative carrying BlaR1 Y^272^A mutation	This study
pDML1279	pDML995 derivative carrying BlaR1 E^213^D mutation	This study
pDML1280	pDML995 derivative carrying BlaR1 E^213^Q mutation	This study
pDML3045	pDML995 derivative carrying BlaR1 R^304^A/R^305^A mutation	This study
pDML1283	pCR-ScriptTMSK(+) derivative carrying BlaR1 L3 loop coding sequence	This study
pDML1284	pDML1283 derivative carrying BlaR1 L3 E^213^A mutation	This study
pDML1285	pDML1283 derivative carrying BlaR1 L3 H^212^A/H^216^A mutation	This study
pDML1286	pDML1283 derivative carrying BlaR1 L3 D^257^A mutation	This study
pDML1287	pDML1283 derivative carrying BlaR1 L3 D^221^A mutation	This study
pDML1293	pDML1283 derivative carrying BlaR1 L3 E^253^A mutation	This study
pDML1288	pLEX derivative allowing the expression of BlaR1 L3 loop	This study
pDML1289	pLEX derivative allowing the expression of BlaR1 L3 carrying E^213^A mutation	This study
pDML1290	pLEX derivative allowing the expression of BlaR1 L3 carrying H^212^A/H^216^A mutation	This study
pDML1291	pLEX derivative allowing the expression of BlaR1 L3 carrying D^257^A mutation	This study
pDML1292	pLEX derivative allowing the expression of BlaR1 L3 carrying D^221^A mutation	This study
pDML1294	pLEX derivative allowing the expression of BlaR1 L3 carrying E^253^A mutation	This study

Ap^r^: ampicillin resistance.

Cm^r^: chloramphenicol resistance.

In order to determine the importance of the HEXXH motif in L3 activity, alanine scanning mutagenesis was used to generate the following mutants: H^212^A/E^213^A/H^216^A, E^213^A, H^212^A/H^216^A and H^216^A. Analyses of these mutants indicate that all of these substitutions give rise to a non-inducible phenotype (Table 1) with a β-lactamase production similar to that obtained for a non-induced wild type strain (data not shown).

Neutral Zinc proteases catalyse cleavage of peptide bonds via a general-base type mechanism in which the glutamate residue of the conserved motif acts as catalytic base. The mutation of this glutamate residue to aspartate (E^213^D) or to glutamine (E^213^Q) prevents β-lactamase induction (Table 1, Figure 3). These results demonstrate that all the residues of this conserved motif play an essential role in the induction process.

The sequence alignment of the protein BlaR1 from *B. licheniformis* with the proteins BlaR1 and MecR from *S. aureus*, highlights four conserved residues that could act as the third zinc ligand (D^221^, E^253^, D^257^and E^274^; see above and Figure 2). Site-directed mutagenesis of these residues in alanine has been done and the resulting plasmids carrying these mutations (pDML1273-74-75-77) were introduced in *B. subtilis* 168 to probe the β-lactamase induction (Table 2). The D^221^, E^253^ and D^257^A mutants show a non-inducible phenotype while the E^274^A mutant retains an inducible phenotype similar to the wild type receptor (Table 1, Figure 3). This result indicates that E^274^ cannot be the third zinc ligand. The three other conserved residues could play an important role either in the enzymatic activity, in the Zinc coordination and/or in the folding of BlaR1 L3 loop. Finally, the mutation of the conserved tyrosine residue in alanine (Y^272^A) also leads to the loss of the β-lactamase induction (Table 1, Figure 3) suggesting that this residue could be part of the relay system involved in the acid-base catalysis.

### L3 Overexpression and Zinc-binding Analysis

**Figure 4:**
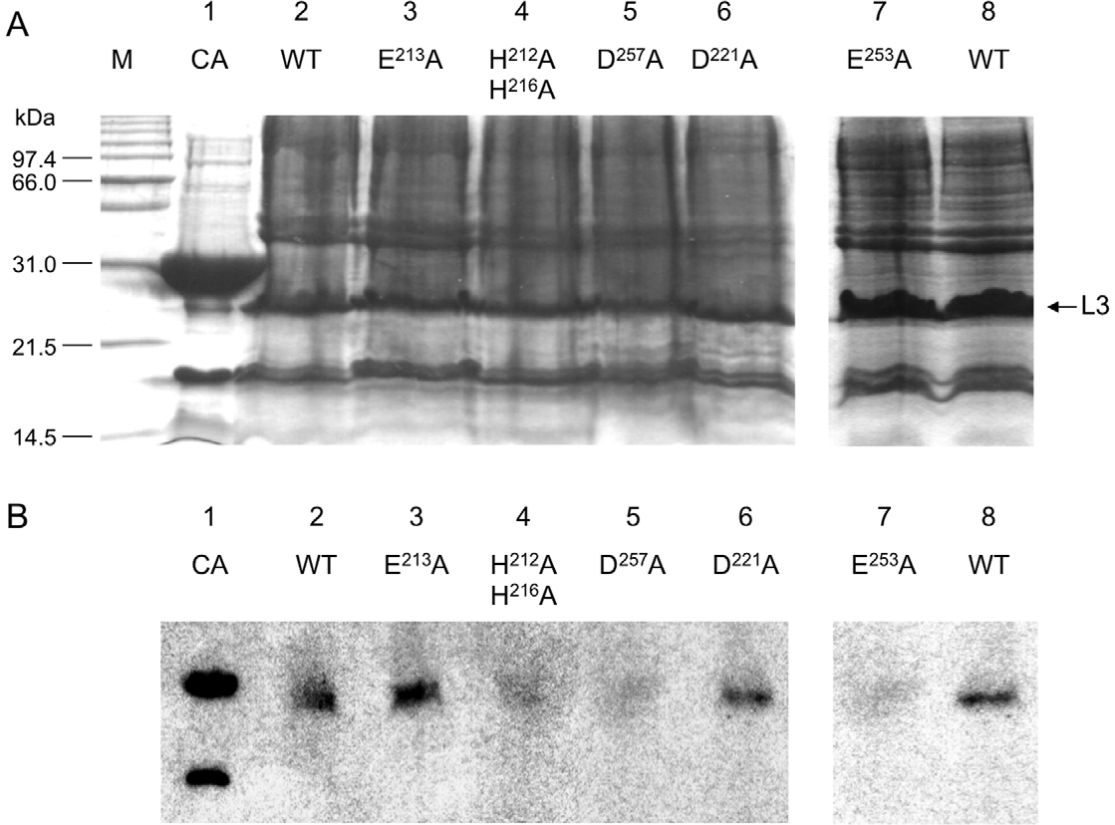
Coomassie Blue-stained SDS-PAGE of partially purified inclusion bodies of wild-type and L3 loop mutants (A) and Zinc blot analysis (B). In A and B: 1: bovine carbonic anhydrase (AC) (∼30 kDa; 5 µg), was used as positive control and 2 to 6 are, respectively, wild type (WT), E^213^A, H^212^A/H^216^A, D^257^ A, D^221^ A and E^253^ A L3 loop mutants. For each gel 40 µg of inclusion bodies were loaded. The fluorographies were exposed at -70°C for 72 hours. M: molecular weight marker. The arrow indicates L3 loops.

The DNA sequence coding for the *B. licheniformis* L3 loop was introduced into the pLex vector in which *l3* was under the control of the inducible P_L_ promoter from lambda phage. The resulting plasmid, pDML1288 (Table 2) was introduced in *E. coli* GI724. In the induced recombinant strain, the L3 loop was overproduced as inclusions bodies. After partial purification and SDS solubilization of inclusion bodies, proteins were separated by SDS-PAGE (Figure 4 A) and transferred onto a nitrocellulose membrane. The blotted proteins were refolded in presence of radioactive zinc chloride solution and a specific radioactive band of 25 kDa was highlighted by autoradiography (Figure 4 B, lane 2). In the utilized technique, it is well accepted that, after protein refolding, a fraction of enzyme macromolecules are correctly folded and active. This band is compatible with the expected molecular mass of L3 (22.116 kDa). The recombinant strains harboring plasmids with mutated *l3* gene encoding for H^212^A/H^216^A, E^213^A, D^221^A, E^253^A or D^257^A (Table 2) has been also analyzed by Zinc blot analysis after solubilization of the inclusion bodies (Figure 4 B). As shown in the Figure 4 B, the H^212^A/H^216^A, E^253^A and D^257^A have lost their ability to bind Zn^++^ whereas E^213^A and D^221^A could be detected as zinc-binding proteins, highlighting the role of H^212^, H^216^, E^253^A and D^257^ residues in Zn binding.

### B. licheniformis L3 is Activated by Autocleavage

**Figure 5:**
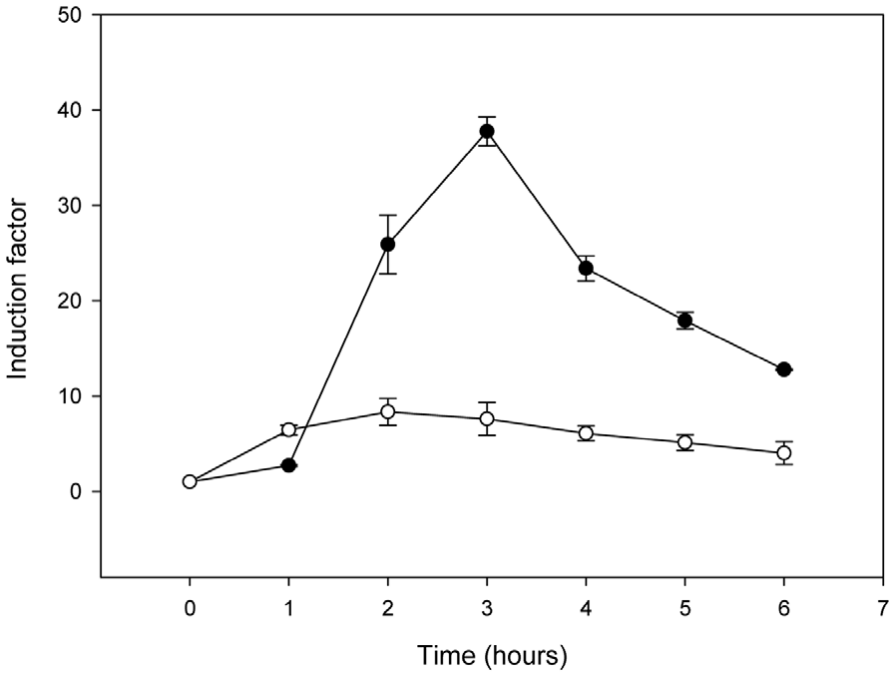
Induction of β-lactamase synthesis in presence (induced) or not (non-induced) of inducer (2.5 µg ml^−1^ cephalosporin C) for the *B. subtilis* strains transformed with pDML995 (wild-type divergeon; •) or pDML3045 (mutation R^304^A/R^305^A;○). The level of the induction is expressed as an induction factor (IF, the ratio between the β-lactamase quantity/A^600^ for induced culture and the β-lactamase quantity/A^600^ for uninduced culture).

**Figure 6:**
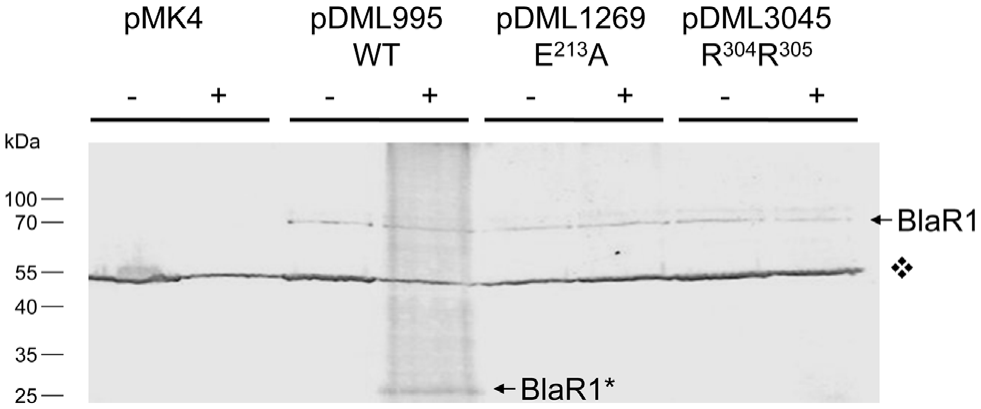
Western blot analysis of membranes of *B. subtilis* transformed with pMK4 (negative control), pDML995 (wild type), pDML1269 (E^213^A mutant) or pDML3045 (R^304^A/R^305^A mutant). Membrane proteins from induced (+) or uninduced (−) cultures were separatedon on SDS-PAGE. Purified BlaR-CTD antibodies were used for the Western blotting. Pre-stained protein molecular weight markers were used (M). The 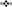 indicates an intense and non-specific band. BlaR1 and BlaR1* highlight, respectively, the full size and the activated *B. licheniformis* BlaR1 receptor.

To avoid inclusion bodies and/or misfolded proteins due to overexpression of recombinant membrane protein, the fate of BlaR1 in presence or not of β-lactam (respectively induced or uninduced) was carried out by Western blot analysis of *B. subtilis-*pDML995 membrane fractions with purified anti-BlaR-CTD antibodies. These experiments revealed the presence of two induced bands: a major band of 33 kDa and a minor one of 68 kDa (Figure 5). The band of low molecular mass is compatible with the cleaved and activated BlaR1 C-terminal domain whereas the other of high molecular mass should be the full-length receptor. These results are in agreement with a cleavage of *B. licheniformis* BlaR1 between the R^304^ and R^305^ residues as in the case of *S. aureus* BlaR1 cleavage between R^294^ and R^295^ (Figure 2, [19]). To confirm these results in *B. licheniformis* BlaR1, the putative residues of the cleavage site has been mutated to R^304^A/R^305^A and the modified receptor was assayed for its ability to induce BlaP production. In presence of β-lactam antibiotic, R^304^A/R^305^A mutants lightly induces BlaP β-lactamase (IF = 7.6, Table 1 and Figure 5). Furthermore, Western blot analysis of membrane fractions from non- and induced cells with purified anti-BlaR-CTD antibodies did not show any L3 cleavage (Figure 6). To explore if the cleavage of BlaR1 observed in induced *B. subtilis*/pDML995 could be the result of L3 proteolytic activity, we analyzed the fate of BlaR1 HA^213^XXH in induced *B. subtilis*/pDML1269 membrane fractions by Western blotting as described above. In this mutant, the catalytic activity of L3 loop is abolished due to the mutation of the conserved glutamic residue of the HEXXH motif involved in the neutral Zinc metallopeptidase family acid-base relay. As shown in Figure 6, no cleavage of BlaR1 HA^213^XXH could be detected in presence of the inducer suggesting that the receptor should be activated via self-proteolysis.

## Discussion

Structural studies on thermolysin and homologous enzymes (Thermolysin-like proteinases, TLPs) have revealed two short conserved motifs in their active sites: HEXXH and EXXXD [25]. The two histidine residues of the HEXXH sequence serve as ligands to zinc, whereas the glutamic acid is involved in the catalytic mechanism by transferring hydrogen atoms and polarizing a zinc-bound water molecule for nucleophilic attack on the scissile peptide bond of the bound substrate. The third zinc ligand is the glutamic acid residue of the sequence motif EXXXD located 20–60 residues downstream of the second histidine of the first motif. For this reason, members of thermolysin enzyme family are named gluzincins [26], [27]. The second histidine residue of the HEXXH motif was found to bridge both the zinc ion and the carboxylate side chain of the aspartic acid residue within the second conserved motif. The exact role of this conserved triad, Zn-His-Asp, is not well understood but is crucial for the catalytic activity [26], [27]. In the absence of a substrate, a water molecule acts as an additional fourth ligand to zinc and is clamped between the catalytic glutamic acid residue of the HEXXH motif and the metal. During TLP catalysis, the catalytic glutamic acid residue accepts a proton from the zinc-bound water nucleophile and transfers it to the leaving group of the substrate. Further stabilization of the transition state is provided by the side-chain of a conserved histidine residue (H^231^ in thermolysin) [28].

Site-directed mutagenesis of selected conserved residues in BlaR1/MecR L3 loops postulated to be involved in catalysis or zinc chelation, combined with zinc-blot experiments and β-lactamase induction assay have confirmed all the features present in the active sites of TLPs. We show that the two histidine residues of the motif H^212^EXXH (*B. licheniformis* BlaR1 numbering) effectively coordinate a Zinc ion and are essential for cytoplasmic signalling. The observed non-inducible β-lactamase phenotype, the conservation of the Zinc binding capacity and the loss of self-proteolysis exhibited by H(E^213^A)XXH mutant are in agreement with a glutamic acid residue acting as catalytic residue and a peptidase activity of L3 loop. The E^253^A and D^257^A mutants, which exhibited the same non-inducible phenotype (Table 1), showed that these residues are crucial for the L3 enzymatic activity and that they are good candidates to be included in the second conserved motif of TLPs. In BlaR1/MecR L3 loops, the EXXXD motif is located 37 residues downstream HEXXH motif, a distance similar to that observed in TLPs, which varies from 20 residues in thermolysin to 59 in mammalian neutral endopeptidase. These new observations allow us to classify BlaR1/MecR L3 loops as new members of TLPs or gluzincins. According to the properties of this enzyme family, the glutamic residue of the EXXXD motif is the third zinc ligand and the aspartic acid residue forms a carboxylate-histidine-zinc interaction crucial for TLP activity (see above). Indeed, as shown by 3D structures from several different enzymes included in the TLP family, this hydrogen bonding stabilizes the coordination of the zinc ion by one of the histidine residues of the HEXXH sequence [29]. In rat dipepeptidyl peptidase, the mutation of the EXXXD motif to EXXXA gives rise to the loss of the zinc ion and concomitantly the loss of enzyme activity [26] as it is the case for L3 EXXXA^257^ mutant (figure 4 and Table 1). By analogy, we postulate that the D^257^ residue of L3 loop is involved in zinc binding by orienting the imidazole ring of the H^212^ residue and/or by enhancing the basicity of the histidine-zinc ligand. To complete the zinc-coordination polyhedron, a water molecule, in interaction with the glutamic acid residue of the HEXXH motif, should be the fourth zinc ligand.

The conserved residues, D^221^ and Y^272^ (*B. licheniformis*BlaR1 numbering) are crucial for the penicillin receptor signalling. The most likely role for Y^272^ is to be involved in the stabilization of the tetrahedral intermediate complex during the catalysis of the activated BlaR1/MecR L3 loop. Indeed, carboxypeptidase A, which shares a common topology with thermolysin for its active site, possesses anY^248^ positioned at the same spatial position of H^231^ in thermolysin [30]. As D^221^ side chain is negatively charged, it is difficult to postulate that this side chain could directly interact with the negatively charged tetrahedral intermediate. Another hypothesis is that this conserved aspartic acid could be essential for substrate binding. As our results are consistent with a self-activation and as the cleavage site includes at least the positively charged side chain of one arginine residue, this hypothesis is plausible.

Similar to many other proteolytic enzymes, TLPs are synthesized as precursors with a propeptide. Up to 70% of known TLPs possess a propeptide containing two distinguished domains: the first one is an intramolecular chaperone (FTP domain) essential for the folding of the catalytic domain whereas the second one (PepSY domain) acts as an inhibitor to prevent detrimental TLP activity [31]. The cleavage of the propeptide occurs through autoprocessing and is mediated by the propeptide [26]. In some TLPs, C-terminal extensions, fused to the Zinc catalytic domain, mediate their binding to insoluble substrates. These C-terminal extensions can be autoprocessed and their cleavage modulates the enzyme specificity and activity [32].

BlaR1/MecR L3 loop activation results in a cascade of events originated by the acylation of the C-teminal sensor by the penicillin and finishing by selfproteolysis of the TLP receptor domain. It has been postulated that the cleavage of the L3 domain is triggered by a conformational change of the transmembrane segments due to a change in the interaction between BlaR/MecR-CTD and BlaR/MecR-NTD in presence of penicillin [16]. The results presented in this paper seem to show that propeptide inhibiting the BlaR1/MecR TLP domain is present and would be located at the C-terminal end of TLP domain. The cleavage site should be included in the K^303^(E/R)↓R conserved motif (Figure 2), in which “↓” indicates the cleaved peptide bond, instead of R^304^↓R motif as previously described [19]. The presence of a C-terminal propeptide inhibiting L3 TLP activity and cleaved by self-proteolysis to give the fully active L3 TLP would be unique to BlaR1/MecR in the TLP superfamily.

The RR/AA cleavage site mutant also sheds new light on the BlaR1/MecR intramolecular transduction mechanism. Indeed, in presence of β-lactam antibiotic, the acylation of the mutated BlaR1 receptor results in a slight induction of BlaP β-lactamase production (about 20% of the wild type) but without L3 cleavage. In the uncleaved protein, the C-terminal portion of the L3 loop acts as an inhibitor of its TLP domain. Cleavage of the KR↓R (both BlaR1 proteins) or KE↓R (*S. aureus*MecR) results in an activation of the peptidase activity, but it is not known whether the C-terminal peptide remains associated or not with the active domain. In fact, in this mutant, we have decoupled the L3 activation due to BlaR-CTD acylation from that caused by L3 selfproteolysis. The BlaP induction results indicate that in this case, the uncleaved L3 loop exhibits a residual activity which seems unable to perform a successful autocleavage but is able to produce a sufficient amount of coactivator to partially inactivate BlaI. This finding is in agreement with a selfproteolysis mechanism in which it is necessary that BlaR-CTD acylation slightly activates L3 loop to allow its own proteolysis and its full activation.

In conclusion BlaR1/MecR L3 loop is a gluzincin and a new member of the TLP superfamily that has diverged very early from canonical TLPs, since no significant identity can be detected between BlaR1/MecR L3 loops and TLPs by using comparison algorithms such as BLAST. The L3 signalling activity of BlaR1/MecR receptor would be repressed by the combination of two intramolecular interactions: one between L3 and its C-terminal propeptide and the second between L2 and BlaR-CTD. Similarly the full L3 activation triggered BlaR-CTD acylation would be the consequence of two successive events described above.

## Materials and Methods

### Bacterial Strains, Plasmids and DNA Manipulations

The strains and plasmids used in this study are described in Table 2. To introduce point mutations into BlaR1 or L3 loop, mutagenesis was carried out using the Quick Change Site-Directed mutagenesis procedure (Stratagene). For all mutations, except BlaR1 R^304^A/R^305^A mutation, the plasmids pDML1251 and 1283 were used as the templates in polymerase chain reactions (PCR) for *blaR1* and l3 loop site-directed mutagenesis, respectively. For BlaR1 R^304^A/R^305^A mutation, pDML995 was used as template. Synthetic oligonucleotides 23–35 bases long containing a mutated codon in the middle of their sequences (Table 3) were employed to mutagenize the original codons. Following the verification of mutations by DNA sequencing (GIGA-DNA sequencing plateform, University of Liège), the corresponding restricted fragments (blaR1: 672 bp SstI-EcoRI; l3 loop: 567 bp NdeI-SalI) were cloned into pDML995 and pLex, respectively.

**Table 3:** Oligonucleotides used in this study.

Name	Nucleotide sequence	Utilities
L3up	5′-TAACATATGGATCCTAGCAATCTAAAAATCGGC-3′	L3 loop sequence
L3rp	5′-AAGCTTGTCGACTTATTTCGCCTTTAGCAAAGGTG-3′	amplification
E213Aup	5′-GCTTCATG**C**ACTGTACCATTGCAAAC-3′	Mutation
E213Arp	5′-GTTTGCAATGGTACAGT**G**CATGAAGC-3′	E^213^A
H212AH216Aup	5′-GAAATGTGTTTTGCTT**GC** TGAACTGTAC**GC** TTGC-3′	Mutation
H212AH216Arp	5′-GCAA**GC**GTACAGTTCA**GC**AAGCAAAACACATTTC-3′	H^212^A/H^216^A
H212AE213AH216Aup	5′-GCTT**GC** TG**C**ACTGTAC**GC** TTGCCAAACG-3′	Mutation
H212AE213AH216Arp	5′-CGTTTGGCAA**GC**GTACAGT**G**CA**GC**AAGC-3′	H^212^A/E^213^A/H^212^A
E253Aup	5′-CGAAAACGGAGATGG**C**GATTTCTTGC-3′	Mutation
E253Arp	5′-GCAAGAAATC**G**CCATCTCCGTTTTCG -3′	E^253^A
D257Aup	5′-GATTTCTTGCG**C**CTTTGCCGTATTAAAAAC-3′	Mutation
D257Arp	5′-GTTTTTAATACGGCAAAG**G**CGCAAGAAATC-3′	D^257^A
D221Aup	5′-GCAAACGAAAAG**CC**ATGCTTATCAAC-3′	Mutation
D221Arp	5′-GTTGATAAGCAT**GG** CTTTTCGTTTGC-3′	D^221^A
Y272Aup	5′-GCACCTCAAA**GC** TGGCGAGGTG-3′	Mutation
Y272Arp	5′-CACCTCGCCA**GC**TTTGAGGTGC-3′	Y^272^A
E274Aup	5′-CCTCAAATATGGCG**C**GGTGATTTTAAAATTCAC-3′	Mutation
E274Arp	5′-GTGAATTTTAAAATCACC**G**CGCCATATTTGAGG-3′	E^274^A
E213Qup	5′-GTTTTGCTTCAT**C** A**G**CTGTACCATTGC-3′	Mutation
E213Qrp	5′-GCAATGGTACAG**C** T**G**ATGAAGCAAAAC-3′	E^213^Q
E213Dup	5′-GTTTTGCTTCATGA**C**CTGTACCATTGC-3′	Mutation
E213Drp	5′-GCAAATGGTACAG**G** TCATGAAGCAAAAC-3'	E^213^D
R304AR305Aup	5'-TACAAACATATCAAA**GC** A**GC**GATTGTTACAGTTGTCAAC-3'	Mutation
R304AR305Arp	5'-GTTGACAACTGTAACAATC**GC**T**GC**TTTGATATGTTTGTA-3'	R^304^A/R^305^A

Modified codons are underlined and mutagenised bases are highlighted.

All routine DNA isolation and manipulation were performed as described by Sambrook et al. [33] or following instructions of the manufacturer. Restriction enzymes and mini-prep kit to prepare small-scale plasmid DNA were purchased from Promega and Fermentas respectively.

### Overexpression of B. licheniformis BlaR1 L3 Loop and Purification of Inclusion Bodies

The coding sequence corresponding to *B. licheniformis* BlaR1 L3 loop (residues Tyr^134^ to Arg^322^) was amplified by PCR from pDML995 plasmid and with KBJ1 and KBJ2 primers (Table 3) containing respectively restriction sites, *Nde*I and *Sal*I at their 5′-ends. The resulting fragment (567 bp) was cloned into pCR-Script to give pDML1283 plasmid. After purification of the resulting plasmid and the sequencing of the insert to verify its integrity, the plasmid was digested with *Nde*I and *Sal*I and the fragment corresponding to the *B. licheniformis* BlaR1 L3 loop was ligated into the pLex vector digested with the same restriction endonucleases to give pDML1288. The pDML1289-94 plasmids are pDML1288 derivatives carrying respectively the mutation E^213^A, H^212^H/H^216^H, E^253^A, D^257^A or D^221^A (Table 2). These mutations were introduced as described above.

The *E. coli* GI724/pDML1288-94 strains were grown over night at 30°C in RM medium (40 mM Na_2_HPO_4,_ 20 mM KH_2_PO_4,_ 10 mM NaCl, 200 mM NH_4_Cl, 2% casamino acids, 1% glycerol, 1 mM MgCl_2_, 100 µg ml^−1^ ampicillin). The culture was then diluted 100 times in IM medium (40 mM Na_2_HPO_4,_ 20 mM KH_2_PO_4_, 10 mMNaCl, 200 mM NH_4_Cl, 2% casamino acids, 0.5% glucose, 1 mM MgCl_2_, 100 µg ml^−1^ ampicilline) and cultivated at 30°C. When the absorbance at 550 nm reached 0.6, L3 loop expression was induced by both the addition of tryptophan (final concentration: 100 µg ml^−1^) and a temperature shift to 37°C. After 3 hours of induction, the cells were harvested by centrifugation, washed with 50 mM HEPES-NaOH (pH 7.5) and suspended in 50 mM Tris-HCl (pH 7.5). Bacterial cells were disrupted using a Basic Z Model disintegrator (Warwick, UK). After the addition of Benzonase (final concentration: 20 µg ml^−1^) and an incubation of 30 min at 4°C, the insoluble cell fraction containing inclusion bodies, was separated from the soluble fraction by centrifugation at 18000 g for 30 min at 4°C. The insoluble fraction was suspended in 50 mM HEPES-NaOH (pH 7.5), 0.5 mM NaCl, 5 mM DTT. After addition of Triton X100 (final concentration: 0.1%), the suspension was incubated under agitation at room temperature for 1 hour. The inclusion bodies containing the L3 loop were recovered by centrifugation at 18000 g, for 30 min and at 4°C.

### Zinc Binding Assay

Soluble or insoluble Zinc binding proteins were detected by Zinc blot assay [34], [35], [36]. Purified inclusion bodies were solubilized by sodium dodecyl sulfate (SDS, solubilization buffer: Tris-HCl 0.5M pH 6.8) and separated by SDS-PAGE. After electrophoresis, the gel was soaked for one hour at 37°C in 25 mM Tris-HCl (pH 8.3), 190 mM glycine, 0.1% SDS, 5% 2-β-Mercaptoéthanol. The proteins were electrophoretically transferred to a nitrocellulose membrane in 25 mM Tris-HCl (pH 8.3), 190 mM glycine, 0.1% SDS, 20% methanol.

The nitrocellulose membrane was first incubated for 1 hour at room temperature in 10 mM Tris-HCl (pH 7.5) to partially renature blotted proteins. The membrane was then soaked two times, 15 min per soaking, in 50 ml of binding buffer (10 mM Tris-HCl (pH 7.5), 0.1 M KCl) containing 1µCi ^65^ZnCl and washed 30 minutes with two changes of binding buffer. The blot was dried one hour at room temperature and analyzed by autoradiography using X-OMAT™ film (Eastman Kodak Company) and an exposure time of 72 hours at −70°C.

### β-lactamase Induction Assay

*Bacillus subtilis* 168 transformed by pDML995 or derivative plasmids was grown at 37°C in Luria-Bertani (LB) medium containing 7 µg ml^−1^ of chloramphenicol until A^600^ reached 0.2. Then, the culture is divided in two portions, one as a control (non induced cells) and the other for the addition of 5 µg ml^−1^ cephalosporin C (induced cells). In these conditions the concentration of inducer is below its minimal inhibitory concentration: 10 and 20 µg ml^−1^, respectively, for *B. subtilis* recombinant strains exhibiting non- and inducible β-lactamase phenotype (Ana Amoroso, unpublished data).

The incubation was continued and samples were withdrawn at hourly intervals for 6 hours. For each sample, the A^600^ was measured to monitor the cell density as well as the β-lactamase activity. This latter activity was spectrophotometrically determined by using nitrocefin as previously describred [17]. The β-lactamase quantity by cell [E_t_] was calculated by using the following equation: v_0_/(cellular density)  =  ΔA^482^/(Δt. ε. A^600^)  =  (k_cat_ × [E_t_] × [S])/(Km + [S]) were v_0_: the initial velocity; ΔA^482^and Δt are absorbance and time variations, respectively; k_cat_: catalytic constant (470 s^−1^) [37]; [S]: substrate concentration (100 µM); Km: 40 µM [37] and ε: nitrocefin coefficient of molar extinction at 482 nm (15000 M^−1^ cm^−1^). The β-lactamase induction level was expressed as “induction factor” (IF), determined as: FI =  ([E_t_] of induced culture)/([E_t_] of uninduced culture).

### Anti-BlaR-CTD Antibodies Production and Western Blotting

A polyclonal antiserum directed against the BlaR1 sensor (BlaR-CTD), was generated by immunizing New Zealand rabbits with purified BlaR-CTD [17]. Polyclonal antibodies were purified against BlaR-CTD immobilized on Sepharose 4B as described in reference [38]. The purified primary antibodies were used in immunoblotting at a final dilution of 1∶1000.

Ten milliliters of induced or uninduced recombinant *B. subtilis* strains were harvested by centrifugation and resuspended in 200 µl of 50 mM Tris-HCl (pH 7.5). Cells were disrupted by sonication in a Branson ultrasonic disintegrator at amplitude of 6 µm for tree 30 s bursts. Membranes fraction, containing the BlaR1 receptor were obtained as insoluble fraction by centrifugation of the lysates. Membrane proteins were solubilized by SDS (solubilization buffer: Tris-HCl 0.5 M pH 6.8) and separated by SDS-PAGE (15% acrylamide) and transferred onto a nitrocellulose membrane (Millipore). Incubation of the membrane with primary antibodies was followed by incubation with alkaline phosphatase-conjugated anti-rabbit antibodies. The immune complexes were detected by a color reaction with nitroblue tetrazolium and 5-bromo-4-chloro-3-indoyl phosphate, as recommended by the manufacturer (Roche Applied Science).
